# Supplementary material for: Optogenetic silencing of hippocampal inputs to the retrosplenial cortex causes a prolonged disruption of spatial working memory
Source: eLife. 2025 Aug 28;13:RP96515. doi: 10.7554/eLife.96515 (PMC12393878; doi:10.7554/eLife.96515)
Supplement: Supplementary file 1. [file elife-96515-supp1.docx]

**SUPPLEMENTARY FILE 1**

**Supplementary file 1a**

**Generalized Linear Mixed Model analysis comparing performance between eArch+ and CTRL, observed in baseline sessions (corresponding to the plots in figure 2C)**

| Model Information |  |
| --- | --- |
| Function | outcome ~ 1 + group + (1\|rat) |
| Link function | Logit |
| Distribution | Binomial |

| Fixed Effects Parameter Estimates | | | | | | | | | | | | | | | | | |
| --- | --- | --- | --- | --- | --- | --- | --- | --- | --- | --- | --- | --- | --- | --- | --- | --- | --- |
|  | | | | | | | | | | **95% Exp(B) Confidence Interval** | | | |  | | | |
| **Names** | **Effect** | | | **Estimate** | | **SE** | | **exp(B)** | | **Lower** | | **Upper** | | **z** | | **p** | |
| (Intercept) |  | (Intercept) |  | 1.653 |  | 0.0761 |  | 5.22 |  | 4.500 |  | 6.06 |  | 21.71 |  | < .001 |  |
| group1 |  | CTRL - ARCH |  | 0.190 |  | 0.1523 |  | 1.21 |  | 0.897 |  | 1.63 |  | 1.25 |  | 0.212 |  |

| Fixed Effect Omnibus tests | | | | | | | |
| --- | --- | --- | --- | --- | --- | --- | --- |
|  | | **X²** | | **df** | | **p** | |
| group |  | 1.56 |  | 1.00 |  | 0.212 |  |

| Post Hoc Comparisons - group | | | | | | | | | | | | | |
| --- | --- | --- | --- | --- | --- | --- | --- | --- | --- | --- | --- | --- | --- |
| **Comparison** | | | | | |  | | | | | | | |
| **group** | |  | | **group** | | **exp(B)** | | **SE** | | **z** | | **p_bonferroni_** | |
| ARCH |  | - |  | CTRL |  | 0.827 |  | 0.126 |  | -1.25 |  | 0.212 |  |
|  | | | | | | | | | | | | | |

**Supplementary file 1b**

**Generalized Linear Mixed Model analysis comparing the ratio of correct trials between eArch+ and CTRL groups, and illuminated and non-illuminated trials (corresponding to the plots in figure 3A and 3B)**

| Model Information |  |
| --- | --- |
| Function | outcome ~ 1 + illumination + group + illumination:group + (1\|rat) |
| Link function | Logit |
| Distribution | Binomial |

| Fixed Effects Parameter Estimates | | | | | | | | | | | | | | | | | |
| --- | --- | --- | --- | --- | --- | --- | --- | --- | --- | --- | --- | --- | --- | --- | --- | --- | --- |
|  | | | | | | | | | | **95% Exp(B) Confidence Interval** | | | |  | | | |
| **Names** | | **Effect** | | **Estimate** | | **SE** | | **exp(B)** | | **Lower** | | **Upper** | | **z** | | **p** | |
| (Intercept) |  | (Intercept) |  | 1.2265 |  | 0.0762 |  | 3.409 |  | 2.936 |  | 3.96 |  | 16.087 |  | < .001 |  |
| illumination1 |  | 1 - 0 |  | 0.0120 |  | 0.0721 |  | 1.012 |  | 0.879 |  | 1.17 |  | 0.166 |  | 0.868 |  |
| group1 |  | CTRL - ARCH |  | 0.4198 |  | 0.1524 |  | 1.522 |  | 1.129 |  | 2.05 |  | 2.755 |  | 0.006 |  |
| illumination1 ✻ group1 |  | 1 - 0 ✻ CTRL - ARCH |  | -0.0547 |  | 0.1442 |  | 0.947 |  | 0.714 |  | 1.26 |  | -0.379 |  | 0.704 |  |
|  | | | | | | | | | | | | | | | | | |

| Fixed Effect Omnibus tests | | | | | | | |
| --- | --- | --- | --- | --- | --- | --- | --- |
|  | | **X²** | | **df** | | **p** | |
| illumination |  | 0.0275 |  | 1.00 |  | 0.868 |  |
| group |  | 7.5923 |  | 1.00 |  | 0.006 |  |
| illumination ✻ group |  | 0.1439 |  | 1.00 |  | 0.704 |  |
|  | | | | | | | |

| Post Hoc Comparisons - group | | | | | | | | | | | | | |
| --- | --- | --- | --- | --- | --- | --- | --- | --- | --- | --- | --- | --- | --- |
| **Comparison** | | | | | |  | | | | | | | |
| **group** | |  | | **group** | | **exp(B)** | | **SE** | | **z** | | **p_bonferroni_** | |
| ARCH |  | - |  | CTRL |  | 0.657 |  | 0.100 |  | -2.76 |  | 0.006 |  |
|  | | | | | | | | | | | | | |

| Post Hoc Comparisons - illumination | | | | | | | | | | | | | |
| --- | --- | --- | --- | --- | --- | --- | --- | --- | --- | --- | --- | --- | --- |
| **Comparison** | | | | | |  | | | | | | | |
| **illumination** | |  | | **illumination** | | **exp(B)** | | **SE** | | **z** | | **p_bonferroni_** | |
| 0 |  | - |  | 1 |  | 0.988 |  | 0.0712 |  | -0.166 |  | 0.868 |  |
|  | | | | | | | | | | | | | |

| Simple effects of group : Parameter estimates | | | | | | | | | | | | | | | | | |
| --- | --- | --- | --- | --- | --- | --- | --- | --- | --- | --- | --- | --- | --- | --- | --- | --- | --- |
| **Moderator levels** | |  | | | | | | | | **95% Exp(B) Confidence Interval** | | | |  | | | |
| **illumination** | | **contrast** | | **Estimate** | | **SE** | | **exp(B)** | | **Lower** | | **Upper** | | **z** | | **p** | |
| 0 |  | CTRL - ARCH |  | 0.447 |  | 0.168 |  | 1.56 |  | 1.12 |  | 2.18 |  | 2.66 |  | 0.008 |  |
| 1 |  | CTRL - ARCH |  | 0.392 |  | 0.169 |  | 1.48 |  | 1.06 |  | 2.06 |  | 2.33 |  | 0.020 |  |
|  | | | | | | | | | | | | | | | | | |

| Simple effects of group : Omnibus Tests | | | | | | | |
| --- | --- | --- | --- | --- | --- | --- | --- |
| **Moderator levels** | |  | | | | | |
| **illumination** | | **X²** | | **df** | | **p** | |
| 0 |  | 7.06 |  | 1.00 |  | 0.008 |  |
| 1 |  | 5.41 |  | 1.00 |  | 0.020 |  |
|  | | | | | | | |

**Supplementary file 1c**

**Generalized Linear Mixed Model analysis comparing the ratio of correct trials between baseline, illuminated and non-illuminated trials, for eArch+ and CTRL groups (corresponding to the plots in figure 3B)**

| Model Information |  |
| --- | --- |
| Function | outcome ~ 1 + illumination_epoch + group + illumination_epoch:group + (1\|rat) |
| Link function | Logit |
| Distribution | Binomial |

Legend of illumination epochs: 0 – non-illuminated, 1 – illuminated trial, 2 - baseline

| Fixed Effects Parameter Estimates | | | | | | | | | | | | | | | | | |
| --- | --- | --- | --- | --- | --- | --- | --- | --- | --- | --- | --- | --- | --- | --- | --- | --- | --- |
|  | | | | | | | | | | **95% Exp(B) Confidence Interval** | | | |  | | | |
| **Names** | | **Effect** | | **Estimate** | | **SE** | **exp(B)** | | | **Lower** | | **Upper** | | **z** | | **p** | |
| (Intercept) |  | (Intercept) |  | 1.3721 |  | 0.0683 |  | 3.944 |  | 3.450 |  | 4.51 |  | 20.090 |  | < .001 |  |
| group1 |  | CTRL - ARCH |  | 0.3431 |  | 0.1364 |  | 1.409 |  | 1.079 |  | 1.84 |  | 2.515 |  | 0.012 |  |
| illum_epoch1 |  | 1 - 0 |  | 0.0120 |  | 0.0720 |  | 1.012 |  | 0.879 |  | 1.17 |  | 0.166 |  | 0.868 |  |
| illum_epoch2 |  | 2 - 0 |  | 0.4562 |  | 0.0917 |  | 1.578 |  | 1.318 |  | 1.89 |  | 4.975 |  | < .001 |  |
| group1 ✻ illum_epoch1 |  | CTRL - ARCH ✻ 1 - 0 |  | -0.0541 |  | 0.1440 |  | 0.947 |  | 0.714 |  | 1.26 |  | -0.376 |  | 0.707 |  |
| group1 ✻ illum_epoch2 |  | CTRL - ARCH ✻ 2 - 0 |  | -0.2785 |  | 0.1833 |  | 0.757 |  | 0.528 |  | 1.08 |  | -1.519 |  | 0.129 |  |
|  | | | | | | | | | | | | | | | | | |

| Fixed Effect Omnibus tests | | | | | | | |
| --- | --- | --- | --- | --- | --- | --- | --- |
|  | | **X²** | | **df** | | **p** | |
| group |  | 6.32 |  | 1.00 |  | 0.012 |  |
| Illum_epoch |  | 28.45 |  | 2.00 |  | < .001 |  |
| group ✻ illum_epoch |  | 2.36 |  | 2.00 |  | 0.307 |  |
|  | | | | | | | |

| Post Hoc Comparisons - group ✻ illumination epoch | | | | | | | | | | | | | | | | | |
| --- | --- | --- | --- | --- | --- | --- | --- | --- | --- | --- | --- | --- | --- | --- | --- | --- | --- |
| **Comparison** | | | | | | | | | |  | | | | | | | |
| **group** | | **Illum_epoch** | |  | | **group** | | **Illum_epoch** | | **exp(B)** | | **SE** | | **z** | | **p_bonferroni_** | |
| ARCH |  | 0 |  | - |  | ARCH |  | 1 |  | 0.962 |  | 0.0942 |  | -0.398 |  | 1.000 |  |
| ARCH |  | 0 |  | - |  | ARCH |  | 2 |  | 0.551 |  | 0.0698 |  | -4.701 |  | < .001 |  |
| ARCH |  | 0 |  | - |  | CTRL |  | 0 |  | 0.635 |  | 0.0984 |  | -2.930 |  | 0.051 |  |
| ARCH |  | 0 |  | - |  | CTRL |  | 1 |  | 0.645 |  | 0.0999 |  | -2.833 |  | 0.069 |  |
| ARCH |  | 0 |  | - |  | CTRL |  | 2 |  | 0.463 |  | 0.0807 |  | -4.419 |  | < .001 |  |
| ARCH |  | 1 |  | - |  | ARCH |  | 2 |  | 0.573 |  | 0.0730 |  | -4.370 |  | < .001 |  |
| ARCH |  | 1 |  | - |  | CTRL |  | 1 |  | 0.670 |  | 0.1042 |  | -2.573 |  | 0.151 |  |
| ARCH |  | 1 |  | - |  | CTRL |  | 2 |  | 0.481 |  | 0.0841 |  | -4.185 |  | < .001 |  |
| ARCH |  | 2 |  | - |  | CTRL |  | 2 |  | 0.839 |  | 0.1615 |  | -0.912 |  | 1.000 |  |
| CTRL |  | 0 |  | - |  | ARCH |  | 1 |  | 1.514 |  | 0.2355 |  | 2.669 |  | 0.114 |  |
| CTRL |  | 0 |  | - |  | ARCH |  | 2 |  | 0.868 |  | 0.1519 |  | -0.808 |  | 1.000 |  |
| CTRL |  | 0 |  | - |  | CTRL |  | 1 |  | 1.015 |  | 0.1071 |  | 0.143 |  | 1.000 |  |
| CTRL |  | 0 |  | - |  | CTRL |  | 2 |  | 0.728 |  | 0.0966 |  | -2.391 |  | 0.252 |  |
| CTRL |  | 1 |  | - |  | ARCH |  | 2 |  | 0.855 |  | 0.1496 |  | -0.894 |  | 1.000 |  |
| CTRL |  | 1 |  | - |  | CTRL |  | 2 |  | 0.717 |  | 0.0951 |  | -2.505 |  | 0.184 |  |
|  | | | | | | | | | | | | | | | | | |

**Supplementary file 1d**

**Generalized Linear Mixed Model analysis comparing the probability of the current trial being correct following an illuminated trial (corresponding to the plots in figure 3C)**

| Model Information |  |
| --- | --- |
| Function | Future_outcome ~ 1 + group + outcome + group:outcome + (1\|rat) |
| Link function | Logit |
| Distribution | Binomial |

| Fixed Effects Parameter Estimates | | | | | | | | | | | | | | | | | |
| --- | --- | --- | --- | --- | --- | --- | --- | --- | --- | --- | --- | --- | --- | --- | --- | --- | --- |
|  | | | | | | | | | | **95% Exp(B) Confidence Interval** | | | |  | | | |
| **Names** | | **Effect** | **Estimate** | | | **SE** | **exp(B)** | | | **Lower** | | **Upper** | | **z** | | **p** | |
| (Intercept) |  | (Intercept) |  | 1.1117 |  | 0.0862 |  | 3.039 |  | 2.567 |  | 3.60 |  | 12.9022 |  | < .001 |  |
| group1 |  | CTRL - ARCH |  | 0.4175 |  | 0.1717 |  | 1.518 |  | 1.084 |  | 2.13 |  | 2.4308 |  | 0.015 |  |
| outcome1 |  | 1 - 0 |  | 0.0539 |  | 0.1219 |  | 1.055 |  | 0.831 |  | 1.34 |  | 0.4425 |  | 0.658 |  |
| group1 ✻ outcome1 |  | CTRL - ARCH ✻ 1 - 0 |  | -0.0215 |  | 0.2431 |  | 0.979 |  | 0.608 |  | 1.58 |  | -0.0885 |  | 0.929 |  |
|  | | | | | | | | | | | | | | | | | |

| Fixed Effect Omnibus tests | | | | | | | |
| --- | --- | --- | --- | --- | --- | --- | --- |
|  | | **X²** | | **df** | | **p** | |
| group |  | 5.90903 |  | 1.00 |  | 0.015 |  |
| outcome |  | 0.19579 |  | 1.00 |  | 0.658 |  |
| group ✻ outcome |  | 0.00783 |  | 1.00 |  | 0.929 |  |
|  | | | | | | | |

| Post Hoc Comparisons - group | | | | | | | | | | | | | |
| --- | --- | --- | --- | --- | --- | --- | --- | --- | --- | --- | --- | --- | --- |
| **Comparison** | | | | | |  | | | | | | | |
| **group** | |  | | **group** | | **exp(B)** | | **SE** | | **z** | | **p_bonferroni_** | |
| ARCH |  | - |  | CTRL |  | 0.659 |  | 0.113 |  | -2.43 |  | 0.015 |  |
|  | | | | | | | | | | | | | |

**Supplementary file 1e**

**Generalized Linear Mixed Model analysis comparing the probability of errors in the current trial following an error trial (corresponding to the plots in figure 4A)**

| Model Information |  |
| --- | --- |
| Function | Errors ~1 + group + illumination + group:illumination + (1\|session_number) + (1\|run_number) |
| Link function | Logit |
| Distribution | Binomial |

| Fixed Effects Parameter Estimates | | | | | | | | | | | | | | | | | |
| --- | --- | --- | --- | --- | --- | --- | --- | --- | --- | --- | --- | --- | --- | --- | --- | --- | --- |
|  | | | | | | | | | | **95% Exp(B) Confidence Interval** | | | |  | | | |
| **Names** | | **Effect** | | **Estimate** | | **SE** | | **exp(B)** | | **Lower** | | **Upper** | | **z** | | **p** | |
| (Intercept) |  | (Intercept) |  | -1.239 |  | 0.107 |  | 0.290 |  | 0.235 |  | 0.357 |  | -11.60 |  | < .001 |  |
| group1 |  | CTRL - ARCH |  | -0.593 |  | 0.159 |  | 0.553 |  | 0.404 |  | 0.755 |  | -3.72 |  | < .001 |  |
| Illumination1 |  | 1 - 0 |  | 0.160 |  | 0.156 |  | 1.173 |  | 0.865 |  | 1.592 |  | 1.03 |  | 0.305 |  |
| group1 ✻ illumination1 |  | CTRL - ARCH ✻ 1 - 0 |  | 0.325 |  | 0.311 |  | 1.384 |  | 0.752 |  | 2.545 |  | 1.04 |  | 0.296 |  |
|  | | | | | | | | | | | | | | | | | |

| Fixed Effect Omnibus tests | | | | | | | |
| --- | --- | --- | --- | --- | --- | --- | --- |
|  | | **X²** | | **df** | | **p** | |
| group |  | 13.84 |  | 1.00 |  | < .001 |  |
| illumination |  | 1.05 |  | 1.00 |  | 0.305 |  |
| group ✻ illumination |  | 1.09 |  | 1.00 |  | 0.296 |  |
|  | | | | | | | |

| Post Hoc Comparisons - group | | | | | | | | | | | | | |
| --- | --- | --- | --- | --- | --- | --- | --- | --- | --- | --- | --- | --- | --- |
| **Comparison** | | | | | |  | | | | | | | |
| **group** | |  | | **group** | | **exp(B)** | | **SE** | | **z** | | **p_bonferroni_** | |
| ARCH |  | - |  | CTRL |  | 1.81 |  | 0.289 |  | 3.72 |  | < .001 |  |
|  | | | | | | | | | | | | | |

**Supplementary file 1f**

**Generalized Linear Mixed Model analysis comparing the probability of errors in the current trial following an error trial, in baseline sessions (corresponding to the plots in figure 4B)**

| Model Information |  |
| --- | --- |
| Function | Errors ~1 + group + (1\|session_number) + (1\|run_number) |
| Link function | Logit |
| Distribution | Binomial |

| Fixed Effects Parameter Estimates | | | | | | | | | | | | | | | | | | | | | | | |  |
| --- | --- | --- | --- | --- | --- | --- | --- | --- | --- | --- | --- | --- | --- | --- | --- | --- | --- | --- | --- | --- | --- | --- | --- | --- |
|  | | | | | | | | | | | | | **95% Exp(B) Confidence Interval** | | | | | |  | | | | |  |
| **Names** | | **Effect** | | **Estimate** | | | **SE** | | **exp(B)** | | | | **Lower** | | | **Upper** | | | **z** | | **p** | | |  |
| (Intercept) |  | (Intercept) |  | | -2.133 |  | | 0.375 | |  | 0.118 |  | | 0.0567 |  | | 0.247 |  | | -5.68 | | < .001 |  | |
| group1 |  | CTRL - ARCH |  | | -0.622 |  | | 0.456 | |  | 0.537 |  | | 0.2197 |  | | 1.311 |  | | -1.37 | | 0.172 |  | |
|  | | | | | | | | | | | | | | | | | | | | | | | |  |

| Fixed Effect Omnibus tests | | | | | | | |
| --- | --- | --- | --- | --- | --- | --- | --- |
|  | | **X²** | | **df** | | **p** | |
| group |  | 1.86 |  | 1.00 |  | 0.172 |  |
|  | | | | | | | |

| Post Hoc Comparisons - group | | | | | | | | | | | | | |
| --- | --- | --- | --- | --- | --- | --- | --- | --- | --- | --- | --- | --- | --- |
| **Comparison** | | | | | |  | | | | | | | |
| **group** | |  | | **group** | | **exp(B)** | | **SE** | | **z** | | **p_bonferroni_** | |
| ARCH |  | - |  | CTRL |  | 1.86 |  | 0.849 |  | 1.37 |  | 0.172 |  |
|  | | | | | | | | | | | | | |

**Supplementary file 1g**

**Generalized Linear Mixed Model analysis comparing the probability of correct trials following illumination of trial_T-n_ (corresponding to the plots in figure 5A)**

| Model Information |  |
| --- | --- |
| Function | trial_n-x_ ~1 + group + (1\|rat) |
| Link function | Logit |
| Distribution | Binomial |

|  | Fixed Effects Parameter Estimates | | | | | | | | | | | | | | | | | |
| --- | --- | --- | --- | --- | --- | --- | --- | --- | --- | --- | --- | --- | --- | --- | --- | --- | --- | --- |
|  |  | | | | | | | | | | **95% Exp(B) Confidence Interval** | | | |  | | | |
|  | **Names** | | **Effect** | | **Estimate** | | **SE** | | **exp(B)** | | **Lower** | | **Upper** | | **z** | | **p** | |
| t-1 | (Intercept) |  | (Intercept) |  | 1.137 |  | 0.0795 |  | 3.12 |  | 2.67 |  | 3.64 |  | 14.30 |  | < .001 |  |
|  | group1 |  | ctrl - arch |  | 0.406 |  | 0.1586 |  | 1.50 |  | 1.10 |  | 2.05 |  | 2.56 |  | 0.010 |  |
| t-2 | (Intercept) |  | (Intercept) |  | 1.161 |  | 0.0949 |  | 3.19 |  | 2.65 |  | 3.84 |  | 12.23 |  | < .001 |  |
|  | group1 |  | ctrl - arch |  | 0.403 |  | 0.1894 |  | 1.50 |  | 1.03 |  | 2.17 |  | 2.13 |  | 0.033 |  |
| t-3 | (Intercept) |  | (Intercept) |  | 1.288 |  | 0.0799 |  | 3.63 |  | 3.10 |  | 4.24 |  | 16.12 |  | < .001 |  |
|  | group1 |  | ctrl - arch |  | 0.334 |  | 0.1590 |  | 1.40 |  | 1.02 |  | 1.91 |  | 2.10 |  | 0.036 |  |
| t-4 | (Intercept) |  | (Intercept) |  | 1.122 |  | 0.0939 |  | 3.07 |  | 2.554 |  | 3.69 |  | 11.95 |  | < .001 |  |
|  | group1 |  | ctrl - arch |  | 0.330 |  | 0.1872 |  | 1.39 |  | 0.964 |  | 2.01 |  | 1.76 |  | 0.078 |  |
|  |  | | | | | | | | | | | | | | | | | |

|  | Fixed Effect Omnibus tests | | | | | | | |
| --- | --- | --- | --- | --- | --- | --- | --- | --- |
|  |  | | **X²** | | **df** | | **p** | |
| t-1 | group |  | 6.56 |  | 1.00 |  | 0.010 |  |
| t-2 | group |  | 4.52 |  | 1.00 |  | 0.033 |  |
| t-3 | group |  | 4.42 |  | 1.00 |  | 0.036 |  |
| t-4 | group |  | 3.11 |  | 1.00 |  | 0.078 |  |
|  |  | | | | | | | |

|  | Post Hoc Comparisons - group | | | | | | | | | | | | | | | | | |  |
| --- | --- | --- | --- | --- | --- | --- | --- | --- | --- | --- | --- | --- | --- | --- | --- | --- | --- | --- | --- |
|  | **Comparison** | | | | | |  | | | | | | | | | | | |  |
|  | **group** | |  | | **group** | | **exp(B)** | | | **SE** | | | **z** | | | **p_bonferroni_** | | |  |
| t-1 | arch |  | - |  | ctrl |  | | 0.666 |  | | 0.106 |  | | -2.56 |  | | 0.010 |  | |
| t-2 | arch |  | - |  | ctrl |  | | 0.669 |  | | 0.127 |  | | -2.13 |  | | 0.033 |  | |
| t-3 | arch |  | - |  | ctrl |  | | 0.716 |  | | 0.114 |  | | -2.10 |  | | 0.036 |  | |
| t-4 | arch |  | - |  | ctrl |  | | 0.719 |  | | 0.135 |  | | -1.76 |  | | 0.078 |  | |
|  |  | | | | | | | | | | | | | | | | | |  |

**Supplementary file 1h**

**Generalized Linear Mixed Model analysis comparing the log(time) spent at the choice point (corresponding to the plots in figure 6A)**

| Model Information |  |
| --- | --- |
| Function | Normalized_log(time) ~1 + illumination + group + illumination:group + (1\|rat) |
| Link function | Identity |
| Distribution | Gaussian |

| Fixed Effects Parameter Estimates | | | | | | | | | | | | | | | | | |
| --- | --- | --- | --- | --- | --- | --- | --- | --- | --- | --- | --- | --- | --- | --- | --- | --- | --- |
|  | | | | | | | | **95% Confidence Interval** | | | |  | | | | | |
| **Names** | **Effect** | | | **Estimate** | | **SE** | | **Lower** | | **Upper** | | **df** | | **t** | | **p** | |
| (Intercept) |  | (Intercept) |  | -0.1971 |  | 0.248 |  | -0.683 |  | 0.289 |  | 13.8 |  | -0.795 |  | 0.440 |  |
| group1 |  | CTRL - ARCH |  | 1.2674 |  | 0.496 |  | 0.295 |  | 2.239 |  | 13.8 |  | 2.556 |  | 0.023 |  |
| illumination1 |  | 1 - 0 |  | 0.0468 |  | 0.274 |  | -0.491 |  | 0.585 |  | 4249.3 |  | 0.171 |  | 0.865 |  |
| group1 ✻ illumination 1 |  | CTRL - ARCH ✻ 1 - 0 |  | -0.6153 |  | 0.549 |  | -1.691 |  | 0.460 |  | 4249.3 |  | -1.121 |  | 0.262 |  |
|  | | | | | | | | | | | | | | | | | |

| Fixed Effect Omnibus tests | | | | | | | | | |
| --- | --- | --- | --- | --- | --- | --- | --- | --- | --- |
|  | | **F** | | **Num df** | | **Den df** | | **p** | |
| group |  | 6.5315 |  | 1 |  | 13.8 |  | 0.023 |  |
| illumination |  | 0.0291 |  | 1 |  | 4249.3 |  | 0.865 |  |
| group ✻ illumination |  | 1.2571 |  | 1 |  | 4249.3 |  | 0.262 |  |
| Nota. Satterthwaite method for degrees of freedom | | | | | | | | | |
|  | | | | | | | | | |

 Table 9. Generalized Linear Mixed Model of the log(time) spent at the choice point by illumination and outcome (corresponding to plot of figure 6B)

| Model Information |  |
| --- | --- |
| Function | Normalized_log(time) ~1 + illumination + group + outcome + illumination:group + illumination:outcome + group:outcome + illumination:group:outcome + (1\|rat) |
| Link function | Identity |
| Distribution | Gaussian |

| Fixed Effects Parameter Estimates | | | | | | | | | | | | | | | | | |
| --- | --- | --- | --- | --- | --- | --- | --- | --- | --- | --- | --- | --- | --- | --- | --- | --- | --- |
|  | | | | | | | | **95% Confidence Interval** | | | |  | | | | | |
| **Names** | | **Effect** | | **Estimate** | | **SE** | | **Lower** | | **Upper** | | **df** | | **t** | | **p** | |
| (Intercept) |  | (Intercept) |  | -0.3571 |  | 0.264 |  | -0.874 |  | 0.159 |  | 17.9 |  | -1.3550 |  | 0.192 |  |
| illumination 1 |  | 1 - 0 |  | 0.0277 |  | 0.330 |  | -0.619 |  | 0.675 |  | 4246.3 |  | 0.0840 |  | 0.933 |  |
| group1 |  | CTRL - ARCH |  | 0.9148 |  | 0.527 |  | -0.118 |  | 1.948 |  | 17.9 |  | 1.7355 |  | 0.100 |  |
| outcome1 |  | 1 - 0 |  | 0.5136 |  | 0.332 |  | -0.137 |  | 1.164 |  | 4255.0 |  | 1.5471 |  | 0.122 |  |
| illumination 1 ✻ group1 |  | 1 - 0 ✻ CTRL - ARCH |  | -0.8037 |  | 0.660 |  | -2.098 |  | 0.491 |  | 4246.3 |  | -1.2171 |  | 0.224 |  |
| illumination 1 ✻ outcome1 |  | 1 - 0 ✻ 1 - 0 |  | 0.0323 |  | 0.661 |  | -1.262 |  | 1.327 |  | 4248.6 |  | 0.0489 |  | 0.961 |  |
| group1 ✻ outcome1 |  | CTRL - ARCH ✻ 1 - 0 |  | 1.1575 |  | 0.664 |  | -0.144 |  | 2.459 |  | 4255.0 |  | 1.7432 |  | 0.081 |  |
| illumination1 ✻ group1 ✻ outcome1 |  | 1 - 0 ✻ CTRL - ARCH ✻ 1 - 0 |  | 0.6806 |  | 1.321 |  | -1.909 |  | 3.270 |  | 4248.6 |  | 0.5152 |  | 0.606 |  |
|  | | | | | | | | | | | | | | | | | |

| Fixed Effect Omnibus tests | | | | | | | | | |
| --- | --- | --- | --- | --- | --- | --- | --- | --- | --- |
|  | | **F** | | **Num df** | | **Den df** | | **p** | |
| illumination |  | 0.00705 |  | 1 |  | 4246.3 |  | 0.933 |  |
| group |  | 3.01211 |  | 1 |  | 17.9 |  | 0.100 |  |
| outcome |  | 2.39345 |  | 1 |  | 4255.0 |  | 0.122 |  |
| illumination ✻ group |  | 1.48133 |  | 1 |  | 4246.3 |  | 0.224 |  |
| illumination ✻ outcome |  | 0.00239 |  | 1 |  | 4248.6 |  | 0.961 |  |
| group ✻ outcome |  | 3.03866 |  | 1 |  | 4255.0 |  | 0.081 |  |
| illumination ✻ group ✻ outcome |  | 0.26539 |  | 1 |  | 4248.6 |  | 0.606 |  |
| Nota. Satterthwaite method for degrees of freedom | | | | | | | | | |
|  | | | | | | | | | |

| Simple effects of group : Parameter estimates | | | | | | | | | | | | | | | | | | | | | | |  |
| --- | --- | --- | --- | --- | --- | --- | --- | --- | --- | --- | --- | --- | --- | --- | --- | --- | --- | --- | --- | --- | --- | --- | --- |
| **Moderator levels** | | | |  | | | | | | | **95% Confidence Interval** | | | | |  | | | | | | |  |
| **illum** | | **outcome** | | **contrast** | **Estimate** | | | **SE** | | | **Lower** | | **Upper** | | | **df** | | **t** | | **p** | | |  |
| 0 |  | 0 |  | CTRL - ARCH |  | 0.908 |  | | 0.915 |  | | -0.8992 |  | 2.72 |  | 157.8 |  | 0.992 |  | | 0.323 |  | |
|  |  | 1 |  | CTRL - ARCH |  | 1.725 |  | | 0.603 |  | | 0.4949 |  | 2.96 |  | 30.5 |  | 2.862 |  | | 0.008 |  | |
| 1 |  | 0 |  | CTRL - ARCH |  | -0.236 |  | | 0.927 |  | | -2.0654 |  | 1.59 |  | 166.3 |  | -0.255 |  | | 0.799 |  | |
|  |  | 1 |  | CTRL - ARCH |  | 1.262 |  | | 0.604 |  | | 0.0290 |  | 2.49 |  | 30.9 |  | 2.088 |  | | 0.045 |  | |
|  | | | | | | | | | | | | | | | | | | | | | | |  |

| Simple effects of group : Omnibus Tests | | | | | | | | | | | |
| --- | --- | --- | --- | --- | --- | --- | --- | --- | --- | --- | --- |
| **Moderator levels** | | | |  | | | | | | | |
| **illumination** | | **outcome** | | **F** | | **Num df** | | **Den df** | | **p** | |
| 0 |  | 0 |  | 0.9850 |  | 1.00 |  | 157.8 |  | 0.323 |  |
|  |  | 1 |  | 8.1900 |  | 1.00 |  | 30.5 |  | 0.008 |  |
| 1 |  | 0 |  | 0.0650 |  | 1.00 |  | 166.3 |  | 0.799 |  |
|  |  | 1 |  | 4.3580 |  | 1.00 |  | 30.9 |  | 0.045 |  |
|  | | | | | | | | | | | |
